# 术前病理学诊断在胸腺肿瘤诊疗中的应用

**DOI:** 10.3779/j.issn.1009-3419.2016.07.05

**Published:** 2016-07-20

**Authors:** 杰 岳, 志涛 谷, 振涛 于, 洪典 张, Zhao MA, 媛 刘, 文涛 方, 中国胸腺肿瘤协作组成员

**Affiliations:** 1 300060 天津，天津医科大学附属肿瘤医院食管癌中心 Department of Esophageal Cancer, Tianjin Medical University Cancer Institute and Hospital, National Clinical Research Center of Cancer, Key Laboratory of Cancer Prevention and Therapy of Tianjin City, Tianjin 300060, China; 2 200030 上海，上海交通大学附属上海胸科医院 Department of Thoracic Surgery, Shanghai Chest Hospital, Shanghai Jiao Tong University, Shanghai 200030, China

**Keywords:** 胸腺肿瘤, 病理诊断, 手术, 预后, 活检, Thymoma, Histology, Surgery, Prognosis, Biopsy

## Abstract

**背景与目的:**

探讨术前病理学诊断在胸腺肿瘤诊断和治疗中的价值及其对胸腺肿瘤治疗的影响。

**方法:**

对中国胸腺肿瘤协作组（Chinese Alliance for Research in Thymomas, ChART）收集的1994年-2012年的多中心且具有明确活检状态的胸腺肿瘤患者的临床病理资料进行回顾性分析，探讨术前病理学诊断的应用趋势及其对胸腺肿瘤患者预后的影响。

**结果:**

1, 902例胸腺肿瘤患者中，术前病理学诊断患者336例（17.1%）。近年来术前病理学诊断的比例较前明显增加（*P*=0.008），胸腔镜/纵隔镜/超声内镜下经支气管活检（endobronchial ultrasound, E-BUS）比例较前升高（*P*=0.029）。术前行病理学诊断患者的生存明显差于无病理学诊断患者（*P* < 0.001），术前病理学诊断后的目的与肿瘤的Masaoka分期（*P* < 0.001）、切除程度（*P*=0.025）、病理类型（*P* < 0.001）具有相关性。术前病理学诊断后直接手术患者的生存要明显优于诱导治疗后再手术患者（*P* < 0.001）。

**结论:**

胸腺瘤诊断主要依靠临床及组织学判断，近年来术前病理学诊断在胸腺肿瘤的诊断和治疗中起重要作用；根治性手术切除是胸腺肿瘤的首先治疗手段；术前病理学诊断后直接手术患者的预后要明显优于诱导治疗后患者。

## Abstract (en)

**Background and objective:**

The aim of this study is to investigate the value of pretreatment biopsy for histological diagnosis and induction therapies in the management of locally advanced thymic malignancies.

**Methods:**

The clinical pathological data of patients with thymic tumors in the Chinese Alliance for Research in Thymomas (ChART) who underwent biopsy before treatment from 1994 to December 2012 were retrospectively reviewed. The application trend of preoperative histological diagnosis and its influence on treatment outcome were analyzed.

**Results:**

Of 1, 902 cases of thymic tumors, 336 (17.1%) had undergone biopsy for histological diagnosis before therapeutic decision was decided. In recent years, percentage of pretreatment histological diagnosis significantly increased in the later ten years than the former during the study period (*P*=0.008). There was also a significant increase in thoracoscopy/mediastinoscopy/endobronchial ultrasound (E-BUS) biopsy as compared to open biopsy (*P*=0.029). Survival in Patients with preoperative biopsy for histology had significantly higher stage lesions (*P* < 0.001) and higher grade malignancy (*P* < 0.001), thus a significantly lower complete resection rate (*P* < 0.001) and therefore a significantly worse survival than those without preoperative biopsy (*P*=0.001). In the biopsied 336 patients, those who received upfront surgery had significantly better survival than those received surgery after induction therapy (*P*=0.001). In stage Ⅲ and Ⅳa diseases, the R0 resection rate after induction therapies increased significantly as compared to the surgery upfront cases (65.5% *vs* 46.2%, *P*=0.025). Tumors downstaged after induction had similar outcomes as those having upfront surgery (92.3% *vs* 84.2%, *P*=0.51). However, tumors not downstaged by induction had significantly worse prognosis than those downstaged (*P*=0.004), and fared even worse than those having definitive chemoradiation without surgery (37.2% *vs* 62.4%, *P*=0.216).

**Conclusion:**

It is crucial to get histological diagnosis for thymoma before surgery or adjuvant treatment and minimally invasive biopsy should be undertaken. Although in our study we could not find the benefit of induction chemotherapy before surgery in survival and recurrence rate, it could increase the R0 resection rate compared with direct surgery in late stage (Ⅲ and Ⅳa).

胸腺瘤（thymoma）是起源于胸腺上皮细胞的肿瘤，是前纵隔最常见的原发性肿瘤，约占所有纵隔肿瘤的17%-30%，其中恶性者占50%左右。胸腺瘤一般发展缓慢，自然病史较长，临床上缺乏随机对照研究。目前胸腺瘤主要采用世界卫生组织（World Health Organization, WHO）病理分型^[1]^及Masaoka分期^[2]^。随着影像技术与医疗水平的发展，胸腺瘤的诊断和治疗水平取得一定的进步，尤其是以根治手术为主的综合治疗，延长了患者的平均生存期，降低了术后复发率，更好的改善患者预后^[3, 4]^。本研究就目前中国胸腺肿瘤合作组收集的多中心且具有明确活检状态的胸腺肿瘤患者的临床病理资料进行回顾性分析，探讨术前病理学诊断的应用趋势及其对胸腺肿瘤治疗的价值和意义，以期为临床治疗提供参考。

## 1 资料与方法

### 1.1 一般资料

对1994年-2012年中国胸腺肿瘤协作组（Chinese Alliance for Research in Thymomas, ChART）收集的多中心胸腺肿瘤2, 104例患者的临床病理资料进行回顾性分析，删除活检状态不明确患者的资料202例，最终1, 902例患者纳入研究。分析术前病理学诊断的应用趋势及其对胸腺肿瘤患者预后的影响。其中术前病理学诊断患者336例，男性192例（57.1%），女性144例（42.9%），平均年龄（46.6±14.1）岁。始发症状仅37例伴发肌无力（11%）。

所有胸腺肿瘤组织学分型经有经验的病理科医生复习手术切除标本的HE染色切片，根据最新WHO胸腺肿瘤组织学分型进行分类^[5]^，胸腺上皮肿瘤分为：A型胸腺瘤（髓质型）；B型胸腺瘤（混合型）；B1型胸腺瘤（皮质为主型，器官样）；B2型胸腺瘤（皮质型）；B3型胸腺瘤（鳞状上皮样、分化好的胸腺癌）和C型胸腺瘤。

胸腺瘤按改良Masaoka病理分期法分期^[6]^，Ⅰ期：肉眼下包膜完整且镜下无包膜侵犯；Ⅱ期：肉眼下侵犯纵隔脂肪组织或纵隔胸膜，或镜下侵犯包膜；Ⅲ期：肉眼下侵犯心包、大血管或肺；Ⅳa期：发现胸膜或心包种植；Ⅳb期：淋巴或血道转移。

### 1.2 随访方法

所有患者至确诊之日起，通过患者复诊、电话以及信件的方式进行随访，术后2年内每3个月随访一次，超过2年后每6月随访一次，随访时间截止至2015年9月，本组病例术后随访率 > 95%。

复发定义为纵隔内病灶未控或者出现新病灶，可发生于临近原发病灶纵隔、胸膜、心包或肺等部位。远处转移为远处脏器或淋巴结转移，包括远离原发病灶的肺、胸膜或心包转移。

### 1.3 统计学方法

采用SPSS 17.0统计软件包进行统计学分析，计量资料采用Mean±SD表示，组间比较采用*t*检验；计数资料用百分比表示，组间比较采用χ^2^检验或*Fisher*精确概率法，生存分析采用*Kaplan-Meier*法，采用*Log-rank*法进行统计分析，*P* < 0.05为差异有统计学意义。

## 2 结果

### 2.1 术前病理学诊断情况

本组1, 902例胸腺肿瘤患者，术前行病理学诊断者336例，占17.7%，未行病理学诊断者1, 566例，占82.3%。1994年-2003年间术前病理学诊断30例，占同期的11.8%，2004年-2012年间术前病理学诊断306例，占同期的18.6%，两组比较，差异具有统计学意义（*P*=0.008）。

本组336例术前病理学诊断的患者中，男性192（57.1%）例，女性144（42.9%）例，平均年龄（46.6± 14.1）岁，合并肌无力患者37（11.0%）例。术前病理学诊断方式见表 1，其中穿刺活检157（46.7%）例，前胸壁切口活检129（38.4%）例，胸腔镜/纵隔镜/E-BUS活检50（14.9%）例。进一步分析发现，1994年-2003年间穿刺活检、前胸壁切口活检、胸腔镜/纵隔镜/E-BUS活检占同期的比例分别为56.7%、43.3%、0；2004年-2012年间穿刺活检、前胸壁切口活检、胸腔镜/纵隔镜/E-BUS活检占同期的比例分别为45.8%、37.9%、16.3%，两组比较差异有统计学意义（*P*=0.029）（表 1）。本组患者按WHO（2004）组织学分型进行分类，其中A型16例，AB型49例，B1型37例，B2型39例，B3型52例，C型99例，胸腺神经内分泌肿瘤（neuroendocrine tumors of the thymus, NETT）7例，类型不明确37例（表 2）。

**1:** 活检方式 Approaches for biopsy in different time period

Treatment time interval	*n*	Needle biopsy	Anterior chest wall incision biopsy	Thoracoscope/mediastinoscope/E-BUS biopsy	*P*
Total		157 (46.7%)	129 (38.4%)	50 (14.9%)	0.029
2004-2012	306	140 (45.8%)	116 (37.9%)	50 (16.3%)	
1994-2003	30	17 (56.7%)	13 (43.3%)	0	
E-BUS: endobronchial ultrasound. 注：本表得到版权所有者© 2011-2016 Journal of Thoracic Disease复制许可。					

**2:** 本组患者根据WHO分型分类结果 Histological diagnosis of biopsy according to WHO classification

WHO type	*n*	Percent
A	16	4.8%
AB	49	14.6%
B1	37	11%
B2	39	11.6%
B3	52	15.5%
C	99	29.5%
Carcinoid	7	2.1%
Undefined	37	11%
WHO: World Health Organization. 注：本表得到版权所有者© 2011-2016 Journal of Thoracic Disease复制许可。		

*Kaplan-Meier*生存分析结果显示：直接手术治疗患者的5年、10年总生存（overall survival, OS）分别为89.5%、82.2%，术前病理学检查患者的5年，10年OS分别为79.4%、58.7%，两组比较生存差异有统计学意义（*P* < 0.001）（图 1）。

**1:**
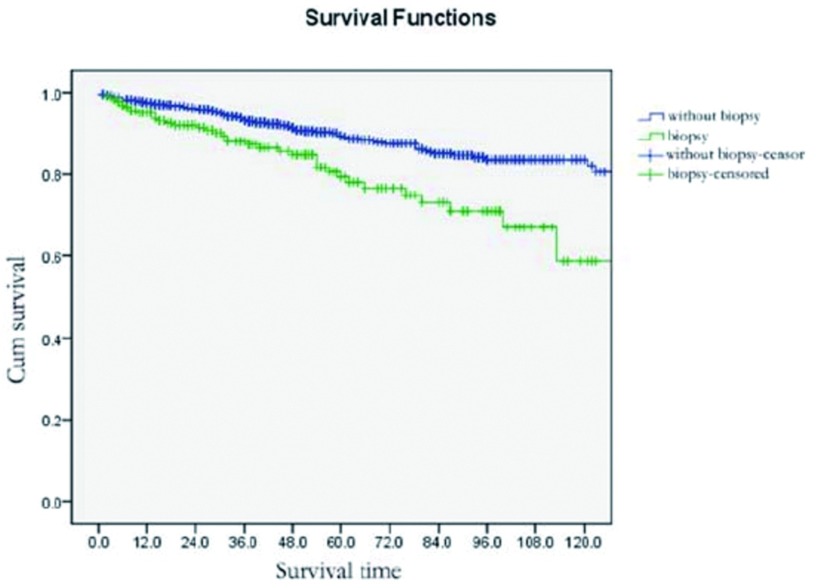
术前病理学诊断对胸腺肿瘤患者预后的影响 Survivals in patients with or without pretreatment biopsy for histological diagnosis

### 2.2 术前病理学诊断目的

术前病理学诊断主要有3个目的：①明确病理类型，排除淋巴瘤等诊断，直接手术；②明确病理类型，选择诱导治疗；③明确病理类型，行根治性放化疗。本组336例术前病理学诊断患者中，明确类型，直接手术患者190例，占总数56.5%；明确类型，术前诱导治疗患者58例，占总数17.3%，明确诊断，根治性放化疗患者88例，占总数26.2%。1994年-2003年间明确类型，直接手术、明确类型，术前诱导治疗、明确诊断，根治性放化疗患者占同期的比例分别为40.0%、36.7%、23.3%；2004年-2012年上述3种活检目的患者占同期的比例分别为58.2%%、15.4%、26.5%，两组比较差异亦具有统计学意义（*P*=0.012）。

### 2.3 术前病理学诊断后直接手术与诱导术前治疗对患者预后的影响

本组336例术前病理学诊断患者中，术前病理学诊断后直接手术患者190例，诱导治疗后手术患者58例。其中18例患者未明确病理分型（直接手术组16例，诱导治疗后手术组2例）。直接手术组患者肿瘤大小为（7.8±3）cm，诱导治疗后患者肿瘤大小为（7.9±2.9）cm，差异无统计学意义（*P*=0.696）。χ^2^检验或*Fisher*精确概率法研究结果显示，两组患者在病理分期、最终病理切除情况及病理类型方面的差异具有统计学意义（*P* < 0.001, *P*=0.016, *P* < 0.001，表 3）。

**3:** 本组患者术前病理学诊断目的与患者临床病理特征的关系 The relationship between preoperative histological diagnosis and clinical pathological characteristics of the patients in this group

Classification	*n*	Purpose of histological diagnosis		*P*
		Directly surgical treatment	Induction therapy	
Tumor size		7.8±3	7.9±2.9	0.696
Masaoka stage				< 0.001
Ⅰ	88	81 (42.6%)	7 (12.1%)	
Ⅱ	32	25 (13.2%)	7 (12.1%)	
Ⅲ	93	60(31.6%)	33 (56.9%)	
Ⅳ	35	24 (12.6%)	11 (19.0%)	
Rresection				0.025
R_0_	178	140 (73.7%)	38 (65.5%)	
R_1_	22	19(10%)	3 (5.2%)	
R_2_	48	31 (16.3%)	17 (29.3%)	
WHO type				< 0.001
A+AB	62	59 (33.9%)	3 (5.4%)	
B1+B2+B3	93	70 (40.2%)	23 (41.1%)	
C+NETT	75	45 (25.9%)	30 (53.6%)	
WHO: World Health Organization; NETT: neuroendocrine tumors of the thymus. 注：本表得到版权所有者© 2011-2016 Journal of Thoracic Disease复制许可。				

鉴于诱导治疗组患者理论上的术前分期均为Ⅲ期+Ⅳ期，所以选择直接手术治疗患者术后分期Ⅲ期+Ⅳ期与所有术前病理学诊断后，诱导治疗患者进行以下比较，结果显示：两组患者在Masaoka分期、最终病理切除情况等方面的差异具有统计学意义（*P* < 0.001，*P*=0.025，表 4）。

**4:** 本组Ⅲ期+Ⅳ期患者术前病理学诊断目的与临床病理特征的关系 The relationship between preoperative histological diagnosis and clinical pathological characteristics of the stage Ⅲ+Ⅳ patients in this group

Classification	*n*	Purpose of histological diagnosis		*P*
		Directly surgical treatment	Induction therapy	
Masaoka stage				0.000
Ⅰ	7	0	7 (12.1%)	
Ⅱ	7	0	7 (12.1%)	
Ⅲ	93	60 (71.4%)	33 (56.9%)	
Ⅳ	35	24 (28.6%)	11 (19.0)	
Rresection				0.025
R0	77	39 (46.2%)	38 (65.5%)	
R1+R2	65	45 (53.6%)	20 (34.5%)	
Pathology				0.095
Thymoma	73	47 (61%)	26 (46.4%)	
Thymic carcinoma	60	30 (39%)	30 (53.6%)	
注：本表得到版权所有者©2011-2016 Journal of Thoracic Disease复制许可。				

*Kaplan-Meier*生存分析结果显示：直接手术治疗患者5年、10年OS分别为84.2%、53%，术前诱导治疗组患者5年、10年OS分别为53.5%、26.2%，差异具有统计学意义（*P*=0.03，图 2）。

**2:**
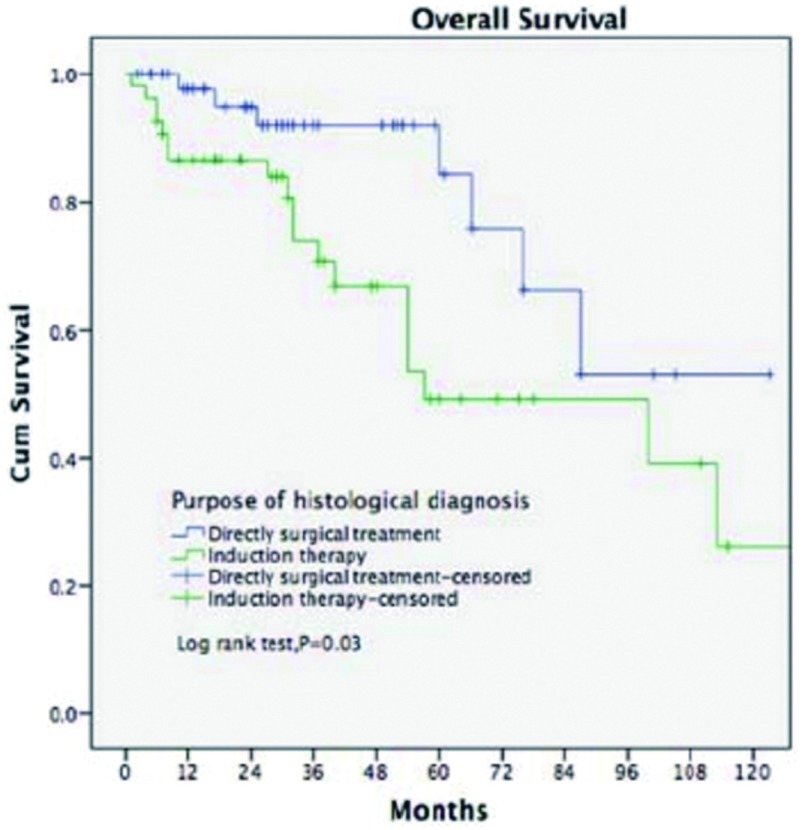
术前病理学诊断目的对本组胸腺肿瘤患者预后的影响 Survivals in patients who had induction therapy or upfront surgery after biopsy

### 2.4 亚组生存分析

为进一步研究不同亚组患者病理学诊断后直接手术与术前诱导治疗后再手术对患者预后的影响，将患者分为不同亚组，结果显示：对R0、R1+R2、胸腺瘤、胸腺癌、Ⅳ期患者术前诱导治疗与直接手术治疗对患者预后的影响，差异无统计学意义，而对于Ⅲ期患者，术前诱导治疗患者的5年生存率为57%，直接手术治疗患者的5年生存率为87%，差异具有统计学意义（*P*=0.003）（表 5）。诱导治疗后未降期的患者5年生存率仅为37.2%，远低于病理学诊断后直接手术的患者（*P*=0.004）；诱导治疗后获得降期的患者5年生存率高达92.3%，与直接手术患者相近（*P*=0.51，图 3）。

**3:**
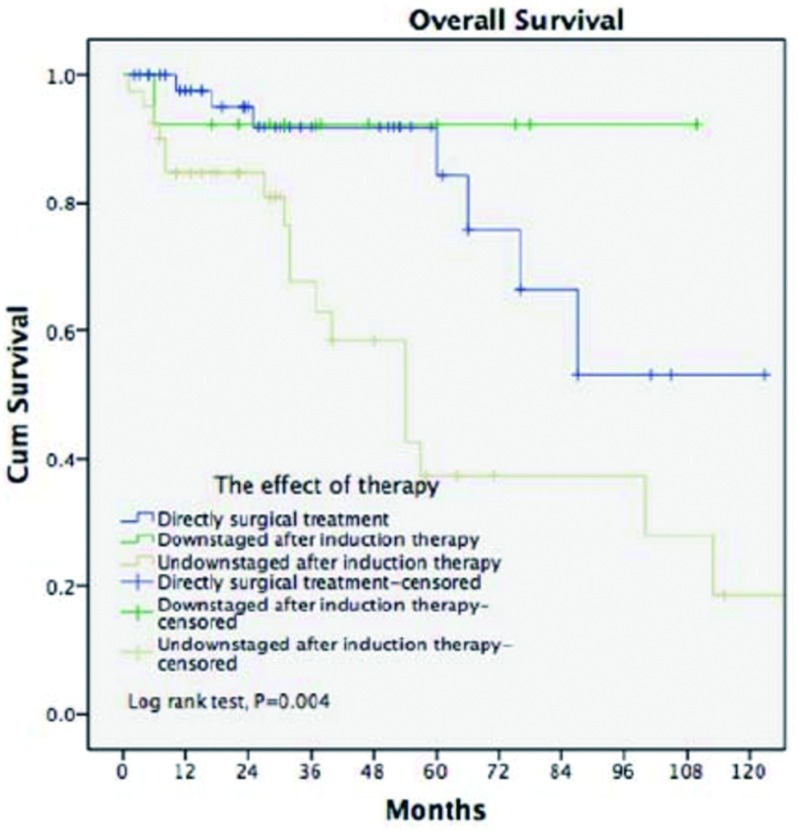
病理学诊断后手术治疗的各亚组生存分析 Survivals in patients who had induction therapy (subgroup) or upfront surgery after biopsy

**5:** 亚组生存分析结果 Subgroup survival analysis

Characteristics	Overall survival (5-year OS)	*P* value
R0		0.127
Preoperative induction therapy	0.557	
Surgical treatment directly	0.72	
R1+R2		0.061
Preoperative induction therapy	0.225	
Surgical treatment directly	0.58	
Thymoma		0.084
Preoperative induction therapy	0.57	
Surgical treatment directly	0.87	
Thymic carcinoma		0.165
Preoperative induction therapy	0.36	
Surgical treatment directly	0.646	
Stage Ⅲ		0.003
Preoperative induction therapy	0.325	
Surgical treatment directly	0.85	
Stage Ⅳ		0.595
Preoperative induction therapy	0	
Surgical treatment directly	0.559	
OS: overall survival. 注：本表得到版权所有者© 2011-2016 Journal of Thoracic Disease复制许可。		

49例患者未接受手术治疗，仅行根治性放化疗，其5年生存率为62.4%，明显低于诱导治疗后获得降期的患者（92.3%），但是高于诱导治疗后未获降期的患者（37.2%）（图 4）。

**4:**
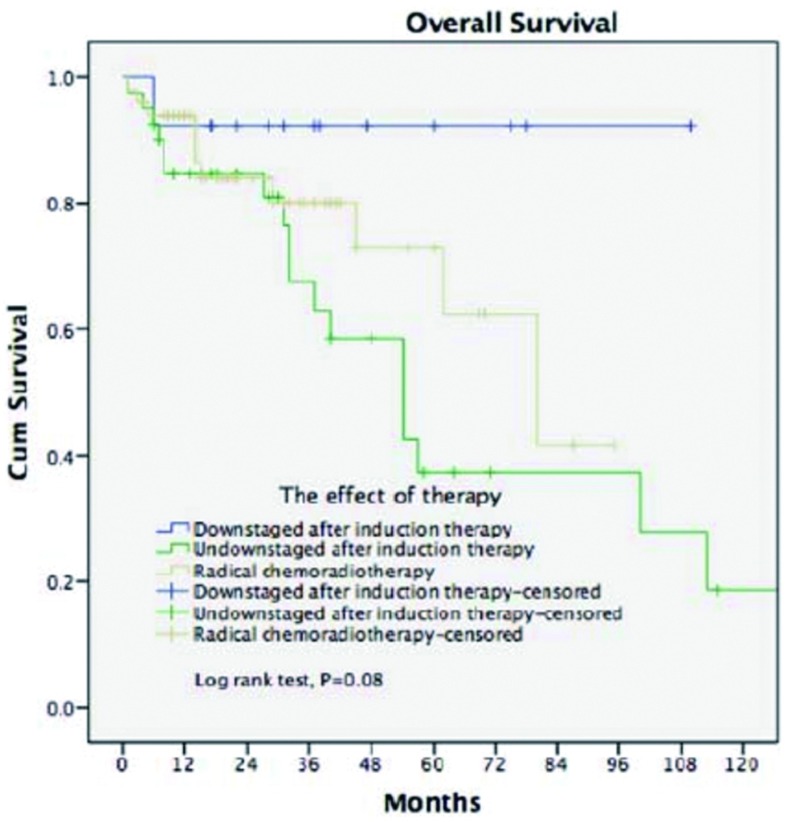
病理学诊断后，诱导治疗的患者与根治性放化疗患者之间的生存分析 Survivals in patients who had induction therapy (subgroup) or radical chemoradiotherapy

## 3 讨论

胸腺瘤是一种较少见的肿瘤，早期多无临床症状，后期可出现咳嗽、胸痛、胸闷等症状。胸腺瘤生物学行为呈隐匿性，多为局部和胸腔内转移，经血行和淋巴转移少见，预后较好^[7, 8]^。外科完整手术切除是治疗侵袭性胸腺瘤的重要手段，除临床证实肿瘤无法切除、存在远处转移或患者条件差不能耐受手术者应尽可能争取手术治疗，它不仅可以直接切除肿瘤，减轻肿瘤负荷消除或缓解临床症状，亦可以提高后续综合治疗的临床效果^[9]^。据文献^[10]^报道，完整切除患者的预后优于其它治疗，包括次全切除联合新辅助或辅助放化疗。手术的彻底程度，组织学分期和病理类型是影响长期生存的重要因素，对于手术未能彻底切除的恶性胸腺瘤，术后给予放、化疗或放疗合并化疗，可以降低复发率，延长生存期^[11]^。对于晚期胸腺瘤患者或不能手术者，放疗能够缩小肿块，从而使患者获取手术机会^[12]^。

WHO组织分型可反映胸腺瘤的生物学行为和临床特征，随着病理类型A型至C型的发展，肿瘤的外侵程度逐渐增强，手术完全切除率也随之降低，基于这种特征，为了提高肿瘤完全切除率，有学者^[13]^提出术前作穿刺活检获得组织学分型诊断，结合影像学表现，做出术前评估。对估计胸腺瘤不能切除的患者，术前给予辅助化疗等综合治疗可以提高其切除率和生存率。

对于获得前纵隔肿物的病理，主要分为两种情况。若肿物体积小，周围侵犯少，则通常是直接进行手术切除，以获取肿瘤组织进行病理诊断。但是由于前纵隔包块与胸主动脉、肺动脉、上腔静脉和心脏等重要器官关系密切，穿刺活检术前需充分了解纵隔与血管、心脏的关系，并排除主动脉瘤的可能，对于解剖结构复杂的较大肿瘤，临床越来越倾向于在影像手段引导下首先进行粗针套管针穿刺活检，获得细胞学和病理学诊断，提高纵隔病变诊断水平，然后根据病理结果并结合影像学特征，制定下一步的诊疗方案^[14]^。计算机断层扫描（computed tomography, CT）有良好的空间分辨率和密度分辨率，可准确显示病灶的大小、形态、位置和病灶内的坏死、空腔区以及病灶与血管等周围结构的解剖关系。因此选择活检导引方法应以CT导引为好，这是目前最为常用的导引手段^[15]^。有相关研究^[16, 17]^表明胸腺瘤CT特征在一定程度上反映了其组织学类型及临床分期。

胸腺穿刺是近年新开展的一种诊疗方法，主要有：①重症肌无力胸腺增生或肿瘤组织的穿刺诊断，穿刺出胸腺组织进病理组织学诊断。②胸腺肿瘤的介入性治疗，经皮穿刺向胸腺肿瘤或增生组织内注入治疗药物，以达到治疗重症肌无力的目的。关于穿刺活检针的选择, 文献报道采用22 G或25G抽吸针，活检正确率分别为91%和88%^[18]^。我们的研究发现ChART收集的多中心胸腺肿瘤患者中，17.1%的患者进行了术前的病理学诊断，并且近年来，术前病理学诊断患者比例较前提高，这充分体现了病理学诊断得到了越来越多的胸外科医生的重视。在众多的R2切除的患者当中，有相当大的比例是仅仅进行了肿物的局部切除或活检，对于患者并没有明显的生存获益。因此，我们强烈建议对于无法完整切除的胸腺瘤患者，应当进行术前病理学诊断活检，明确病理后行放疗或者化疗。另外新增加胸腔镜/纵隔镜/E-BUS活检方式，穿刺活检、前胸壁切口活检比例均下降，体现了胸腔镜/纵隔镜/E-BUS在胸腺瘤诊断中的价值。

胸腺瘤作为放射敏感性肿瘤，放疗的作用已逐步显著，无论是辅助或新辅助放疗在浸润性胸腺癌中的应用是有价值的。但是Ⅲ期患者近20年来总5年生存率未见明显提高，Ⅲ期患者未完全切除即使术后放疗或放化疗预后仍不理想，因此术前新辅助放疗或放化疗可缩小肿瘤体积，降低肿瘤的血液供应，提高手术完整切除率，减少局部和胸膜复发率提高生存率，进而延长患者生存期改善生存质量。关于术前新辅助化疗，Lucchi等^[19]^报道7例Ⅲ期胸腺癌应用顺铂、表阿霉素和足叶乙甙进行新辅助化疗，取得4例CR、3例PR，化疗完成后所有患者进行外科切除和术后放疗，作者认为多学科综合治疗可以改善胸腺癌患者的生存。Venuta等^[20]^报道21例（8例Ⅲ期，13例Ⅳa期）胸腺瘤新辅助化疗前瞻性研究结果，缓解率为100%，Ⅲ期患者8年生存率为92%，Ⅳa期为68%。

许多学者认为手术切除的完整性是可预测肿瘤复发的最重要预后因素。完整手术切除的预后优于次全切除联合辅助或新辅助放疗和化疗^[21]^。本研究结果提示，病理学诊断为直接手术患者的预后要明显优于诱导治疗后再手术患者，该研究结果可能因为术前病理学诊断后，直接手术治疗的患者中包括55.8%的Ⅰ期+Ⅱ期患者，所以手术R0切除率高于诱导治疗组，而诱导治疗组患者理论上的术前分期均为Ⅲ期+Ⅳ期，肿瘤与周围组织关系密切，手术切除率低有关，所以直接手术治疗患者预后要优于术前辅助治疗患者。

由于缺少随机分组研究结果或大样本的回顾性分析结果来明确术后辅助治疗的作用，关于不同分期胸腺瘤的手术后辅助治疗的标准仍存在争议。一项采用美国医疗保险监督、流行病学和最终结果（Surveillance, Epidemiology, and End Results, SEER）登记资料的回顾性研究显示，术后放疗对Ⅰ期胸腺瘤患者无生存获益，但可以显著提高Ⅱ期和Ⅲ期胸腺瘤患者的总生存，特别是对不完全切除的患者^[22]^。一项荟萃分析结果显示，术后放疗对完全切除的Ⅱ期、Ⅲ期胸腺瘤患者无减少局部复发率和提高总生存率的益处^[23]^。然而Rios等^[24]^认为Ⅱ期、Ⅲ期患者无论是否行根治性手术均应行术后辅助治疗。但是，Ⅱ期患者术后放疗虽能显著降低局部复发率，却不能减少胸膜播散的发生，Ⅲ期胸腺瘤R0切除术后，大量研究支持术后放疗。Mangi等^[25]^报道了155例胸腺瘤术后辅助放疗的结果，49例Ⅱ期患者中14例进行了放疗，所有病例均为完整切除，增加术后放疗没有显著改善Ⅱ期胸腺瘤的局部控制率和远地转移率。因此作者认为大多数Ⅱ期胸腺瘤不需要术后放疗，而在完全切除后随访。因此，有关术后辅助治疗对胸腺瘤患者的预后价值还需深入研究。

总之，回顾性总结20年ChART胸腺肿瘤诊断和治疗的经验，结果显示根治性手术切除是胸腺肿瘤治疗的重要手段，术前病理学诊断在胸腺肿瘤的诊治中起重要作用；术前病理学诊断后直接手术患者的预后要明显优于诱导治疗后患者。对于胸腺肿瘤巨大或侵犯主要血管，可能无法完全切除时，应积极行术前新辅助放疗或放化疗，以提高根治术的可能，从而达到较好的疗效。
