# 更正声明

**DOI:** 10.3779/j.issn.1009-3419.2016.09.15

**Published:** 2016-09-20

本刊2016年第19卷第7期刊登的题为“术前病理学诊断在胸腺肿瘤诊疗中的应用”[岳杰，谷志涛，于振涛，张洪典，刘媛，方文涛，中国胸腺肿瘤协作组成员.术前病理学诊断在胸腺肿瘤诊疗中的应用.中国肺癌杂志, 2016, 19(7): 437-444.]一文中：第437页，中文作者应为“岳杰，谷志涛，于振涛，张洪典，马钊，刘媛，方文涛，中国胸腺肿瘤协作组成员”。特此更正。对此深表歉意。

Erratum: Pretreatment Biopsy for Histological Diagnosis and Induction Terapy in Tymic Tumors

Jie YUE, Zhitao GU, Zhentao YU, Hongdian ZHANG, Zhao MA, Yuan LIU, Wentao FANG, Members of the Chinese Alliance for Research in Tymomas

Zhongguo Fei Ai Za Zhi, 2016, 19(7): 437-444.

In the version of this article initially published, error appeared on page 437. Te authors’ Chinese name list should be “岳杰，谷志涛，于振涛，张洪典，马钊，刘媛，方文涛，中国胸腺肿瘤协作组成员”.

## Notes

https://www.ncbi.nlm.nih.gov/pmc/articles/PMC6133982/

